# Advancing stroke rehabilitation: the potential and challenges of closed-loop brain-computer interface technology

**DOI:** 10.3389/fneur.2026.1861673

**Published:** 2026-06-24

**Authors:** Yan Cheng, Xiangkui Guo, Lijia Dong, Qiang Deng, Maoqi Qiu, Zhongchun Luo

**Affiliations:** Department of Rehabilitation Medicine, People’s Hospital of Leshan, Leshan, Sichuan, China

**Keywords:** closed-loop brain-computer interface, cognitive function, motor function, neuroplasticity, stroke rehabilitation

## Abstract

**Background:**

Stroke is one of the leading causes of long-term disability in older worldwide. As an emerging neuromodulation intervention, closed-loop brain-computer interfaces (BCIs) aim to promote the reconstruction of the damaged cortex through real-time feedback mechanisms. This study aims to systematically review the latest clinical advancements, neural mechanisms, and challenges of closed-loop BCIs in post-stroke rehabilitation.

**Methods:**

Following the PRISMA guidelines, this study systematically searched databases including PubMed, Web of Science, Cochrane Library, Embase, Scopus, and IEEE Xplore. Given the high methodological heterogeneity in intervention paradigms and outcome measures across different studies, a qualitative synthesis strategy was employed. The minimal clinically important difference (MCID) was introduced to evaluate the substantive clinical benefits of various interventions. Ultimately, 42 original studies meeting the strict definition of closed-loop systems were included.

**Results:**

Closed-loop BCI technology demonstrates multi-dimensional application potential in stroke rehabilitation. In motor rehabilitation, BCIs combined with external actuators (e.g., robotics, FES) promote interhemispheric functional rebalancing and corticospinal tract remodeling. In the cognitive domain, although neurofeedback has shown initial efficacy in improving specific executive functions and attention, the current evidence exhibits high heterogeneity and requires cautious interpretation. Regarding safety, adverse reactions to non-invasive devices primarily manifest as mild fatigue; for invasive systems, the incidence of device-related adverse events is approximately 5.6 per 1,000 device-days, indicating overall controllable safety.

**Conclusion:**

Closed-loop BCIs provide a promising novel neuromodulation strategy for stroke rehabilitation. Future validation of their efficacy and acceleration of clinical translation will rely on multicenter randomized controlled trials (RCTs), standardized core outcome sets (COS), and deep integration with artificial intelligence (AI).

## 1 Introduction

**Figure 1:**
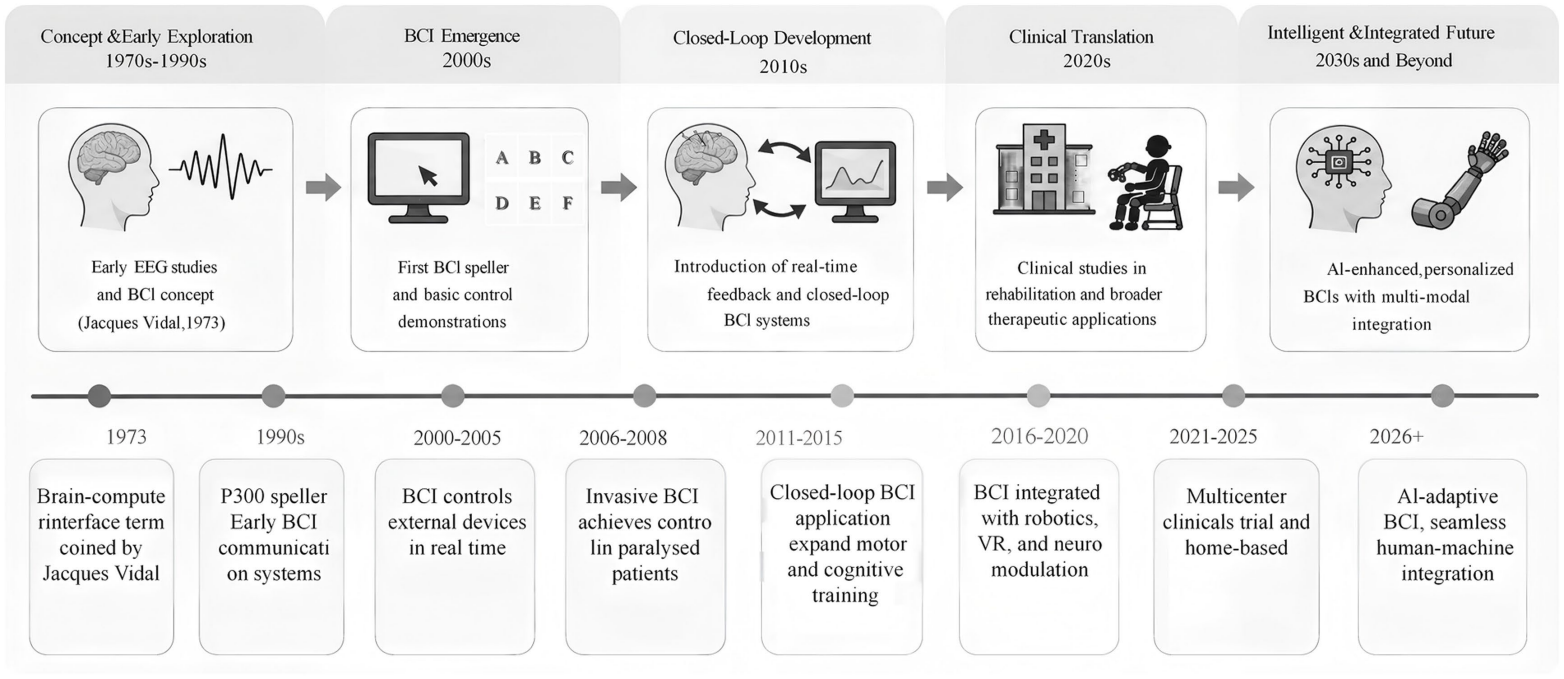
Timeline chart of the development of closed-loop brain-computer interfaces. (1) This schematic illustration depicts the half-century chronological evolution of BCI technology across five distinct developmental epochs: Concept and Early Exploration (1973s–1990s): Initiated by Jacques Vidal coining the term “brain-computer interface” in 1973, early milestones focused on foundational EEG characterization and the development of open-loop communication systems such as the P300 speller. (2) BCI Emergence (2000s): Marked by the realization of real-time control over external devices and the successful proof-of-concept demonstrations of invasive BCIs in paralyzed human cohorts. (3) Closed-Loop Development (2010s): Characterized by the historical shift from unidirectional control to bidirectional neural coupling through the introduction of real-time sensorimotor feedback, which expanded BCI applications into motor and cognitive rehabilitation. (4) Clinical Translation (2020s): Defined by the rigorous validation of BCIs through multi-center randomized controlled trials (RCTs), seamlessly integrating neural guidance with assistive robotics, FES, and virtual reality for both clinical and home-based settings. 5. Intelligent & Integrated Future (2030s and Beyond): Envisions a human-centered ecosystem featuring AI-adaptive decoding pipelines, advanced multi-modal integration (e.g., combining high-density EEG with metabolic neuroimaging), and seamless human-machine co-adaptation. BCI, brain-computer interface; EEG, electroencephalography; FES, functional electrical stimulation; VR, virtual reality; AI, artificial intelligence.

Currently, there are approximately 15 million new incident cases of stroke globally each year, which is a major cause of disability and mortality among the elderly, severely impacting individual quality of life and social healthcare systems (1). While traditional rehabilitation methods are effective to a certain extent, their clinical benefits are relatively limited for patients with severe impairments (2, 3). Closed-loop brain-computer interface (BCI) technology is currently emerging as an interdisciplinary frontier between neurology and rehabilitation medicine. Since Jacques Vidal first coined the term “brain-computer interface” in 1973, BCI technology has developed rapidly, demonstrating tremendous potential, particularly in the field of stroke rehabilitation (4, 5). Distinct from traditional passive interventions, closed-loop BCIs integrate patients’ endogenous motor or cognitive intentions with exogenous real-time sensory feedback, actively participating in driving the remodeling process of functional impairments (see Figure 1).

## 2 Methods

### 2.1 Literature search strategy

This study conducted a comprehensive literature search across databases including PubMed, Web of Science, Cochrane Library, Embase, Scopus, IEEE Xplore, and institutional library databases, covering the period from database inception to December 31, 2024. The search utilized a combination of MeSH terms and text words. The specific search string was as follows: (“stroke” OR “cerebrovascular accident” OR “post-stroke”) AND (“brain–computer interface” OR “BCI” OR “neurofeedback” OR “motor imagery” OR “P300” OR “fNIRS” OR “ECoG”) AND (“closed-loop” OR “real-time” OR “adaptive feedback” OR “contingent stimulation”) AND (“rehabilitation” OR “functional electrical stimulation” OR “robot-assisted” OR “virtual reality”). To encompass post-stroke functional impairments, the following qualifiers were added to sub-domain searches: upper limb functional recovery: (FMA-UE OR ARAT OR WMFT OR MAL); lower limb and walking function: (FMA-LE OR 10MWT OR 6MWD OR BBS OR TUG); cognitive and speech function: (MoCA OR executive function OR attention OR memory OR aphasia OR WAB OR BNT). The inclusion criteria for the literature were: (1) patients explicitly diagnosed with stroke; (2) utilization of a closed-loop BCI system, defined as neural signals driving a feedback device in real-time; (3) clear reporting of rehabilitation-related functional outcomes, including upper limb, lower limb, cognitive, or speech functions; (4) original research articles. The exclusion criteria were: (1) animal experiments or studies involving healthy subjects; (2) utilization of open-loop BCI systems. Following the initial screening of records and abstracts, full-text reviews of the remaining studies were conducted, and the final included studies were determined based on the aforementioned criteria. After screening, 42 original studies that met the requirements were included (see Supplementary Figure S1).

### 2.2 Definition of closed-loop and inclusion/exclusion criteria

To ensure evidence homogeneity, this study established a strict methodological definition for “closed-loop BCI”: within a single trial, brain signals must immediately drive associated feedback or intervention upon online decoding, thereby altering the subject’s behavioral or neural state in real-time. Based on this, specific criteria for excluding borderline “open-loop” systems were as follows: (1) end-to-end latency >1 s or long-latency offline processing inferred from the methodology. (2) continuous feedback update rate <5 Hz or event-triggered evaluation intervals exceeding 2 s. (3) feedback stimulation not determined in real-time by current brain signals (relying solely on time scripts) or offline retraining conducted outside the trial (6–8).

### 2.3 Qualitative synthesis and minimal clinically important difference (MCID)

Due to significant methodological heterogeneity among the studies in outcome assessments (e.g., FMA-UE, ARAT, MAS), closed-loop feedback modalities (FES, robotics, visual feedback), and intervention durations (ranging from 2 to 8 weeks), with most studies only reporting pre- and post-intervention differences, conducting a quantitative meta-analysis could lead to statistical artifacts according to the Cochrane Handbook for Systematic Reviews of Interventions standards (9). Therefore, to scientifically measure the substantive clinical benefits of different interventions, the “Minimal Clinically Important Difference (MCID)” was introduced as the core criterion for determining functional improvement. The MCID determination thresholds were strictly set as follows: FMA-UE ≥ 5 points; ARAT ≥6 points for the sensitive period and ≥12 points for the non-sensitive period; MAS ≥ 0.5 points; BBS ≥ 7 points; MoCA ≥1 point for the sensitive period and ≥2 points for the non-sensitive period (10).

### 2.4 Evidence quality assessment criteria

To objectively evaluate the methodological quality and evidence strength of the studies, this study developed a standardized evidence quality assessment system tailored to the characteristics of closed-loop BCI clinical trials in stroke. Based on study design, sample size, and control settings, the evidence was strictly categorized into four levels: (1) High-strength evidence: randomized controlled trials (RCTs) with adequate sample sizes (*n* ≥ 20), explicit parallel control groups (e.g., standard physical therapy or conventional occupational therapy), and low risk of methodological bias. (2) Moderate-strength evidence: explicit parallel control design, non-randomized controlled trials, sham stimulation/sham BCI controls, incomplete blinding, or relatively small sample sizes (*n* < 20). (3) Preliminary/low-strength evidence: early exploratory clinical studies, before-and-after trials without parallel control groups, or small sample sizes. (4) Very low-strength evidence: case studies or extremely small sample sizes (*n* ≤ 5). Following these quantitative criteria, independent methodological grading and annotations were executed in the “Quality Evaluation” column of Tables 1–3, ensuring objectivity in the qualitative data synthesis.

**Table 1:** Study on upper limb functional rehabilitation after stroke based on closed-loop brain-computer interface.

Study	BCI modality	EG/CG	Experimental intervention	Control intervention	Intervention period	Outcome index	FMA-UE outcome	Quality evaluation
Ang KK 2014	EEG	6/8HK/7SAT	BCI-HK 1 h BCI + 30 min TAAM	HK: 1 h HK + 30 min TAAM; SAT: 1.5 h TAAM	6 weeks, 18 sessions	FMA-UE	5.7 ± 1.7/3.0 ± 2.1/2.0 ± 1.6	Randomized Controlled Trial; Sample size *n* = 14; Evidence strength: high
Ang KK 2015	EEG	11/14	BCI-MIT-Manus robot 1.5 h	MIT-Manus robot 1.5 h	4 weeks, 12 sessions	FMA-UE	6.5 ± 1.4/3.8 ± 1.3	Randomized Controlled Trial; Sample size *n* = 25; Evidence strength: high
Ang KK	EEG	10/9	20 min tDCS + 1 h BCI-MIT-Manus robot	20 min sham tDCS + 1 h sham BCI	2 weeks, 10 sessions	FMA-UE	8.3/4.7	Randomized Controlled Trial; Sample size *n* = 19; Includes sham/BCI control; Evidence strength: moderate
Biasiucci 2018	EEG	14/13	BCI-FES 1 h	Sham-FES 1 h	5 weeks, 10 sessions	FMA-UE, ESS, MRC, MAS	6.6 ± 1.0/0.4 ± 0.9	Randomized Controlled Trial; Sample size *n* = 27; Includes sham/BCI control; Evidence strength: high
Cheng 2020	EEG	5/5	BCI-assisted soft robotic glove 90 min + 30 min SAT	Soft robotic glove 90 min + 30 min SAT	6 weeks, 18 sessions	FMA-UE, ARAT	9.6/4.4	Randomized Controlled Trial; Sample size *n* = 10; Evidence strength: preliminary
Frolov 2017	EEG	30/10	BCI-exoskeleton 30 min + SPT	Sham BCI 30 min	2 weeks, 10 sessions	FMA-UE, ARAT	8.6/2.3	Randomized Controlled Trial; Sample size *n* = 40; Includes sham/BCI control; Evidence strength: high
Kim 2016	EEG	15/15	AOT based BCI-FES 30 min + 30 min Routine training	Routine training 30 min	4 weeks, 12 sessions	FMA-UE, MAL, MBI	14.3/6.2	Randomized Controlled Trial; Sample size *n* = 30; Evidence strength: high
Li 2014	EEG	7/7	BCI-FES 1–1.5 h + CON	FES 20 min + CON	8 weeks, 24 sessions	FMA-UE, ARAT	7.4	Controlled Trial (non-randomized); Sample size *n* = 14; Evidence strength: moderate
Mihara 2013	fNIRS	10/10	BCI-visual feedback 20 min + 120 min NDT	Sham BCI 20 min + 120 min NDT	2 weeks, 6 sessions	FMA-UE, ARAT, MAL	11.2/4.3	Randomized Controlled Trial; Sample size *n* = 20; Includes sham/BCI control; Evidence strength: moderate
Pichiorri 2015	EEG	14/14	BCI-virtual hand 1 h	MI, 1 h	4 weeks, 12 sessions	FMA-UE MAS	16.9/11.1	Controlled Trial (non-randomized); Sample size *n* = 28; Evidence strength: moderate-high
Ramos-Murguialday 2013	EEG	16/16	BCI-orthosis 1 h + 1 h BPT	Sham BCI-orthosis 1 h + 1 h BPT	4 weeks, 20 sessions	FMA-UE GAS, MAL	6.6/1.1	Controlled Trial (non-randomized); Sample size *n* = 32; Includes sham/BCI control; Evidence strength: moderate-high
Wu 2019	EEG	14/11	BCI-exoskeleton 1 h + 1 h routine training	Routine training 2 h	4 weeks, 20 sessions	FMA-UE ARAT, WMFT	8.7	Controlled Trial (non-randomized); Sample size *n* = 25; Evidence strength: moderate
Curado 2015	EEG	16/14	BCI-robotic orthosis 1 h + Behavioral physical therapy 1 h	Sham BCI-robotic orthosis 1 h + Behavioral physical therapy 1 h	4 weeks, 20 sessions	FMA-UE, EMG	None	Controlled Trial (non-randomized); Sample size *n* = 30; Includes sham/BCI control; Evidence strength: moderate
Liao 2023	EEG	20/20	MI-BCI visual and auditory feedback, 45–60 m	Routine rehabilitation training	3 weeks, 15 sessions	FMA-UE MAS HMCAS	9.8/4.2	Controlled Trial (non-randomized); Sample size *n* = 40; Evidence strength: moderate-high
Jiang 2016	EEG	10/10	BCI + FES 20 min	FES 20 min	6 weeks, 30 sessions	MFT MAS	8.5/3.2	Randomized Controlled Trial; Sample size *n* = 20; Evidence strength: moderate
Ramirez-Nava 2023	EEG P300	7/7	BCI-FEST 1 h	Conventional therapy 1 h	4 weeks, 20 sessions	FMA-UE ARAT	8.3	Controlled Trial (non-randomized); Sample size *n* = 14; Evidence strength: preliminary
Brunner 2024	EEG	15/20	BCI + FES 60 min	Standard physiotherapy and occupational therapy	3–4 weeks, 12 sessions	ARAT	4.0/2.8	Randomized Controlled Trial; Sample size *n* = 35; Evidence strength: high
Kim 2025	EEG	12/13	BCI + FES 60 min + Conventional therapy 30 min	Sham BCI + FES 60 min + Conventional therapy 30 min	4 weeks, 20 sessions	FMA-UE MAS WF	7.9/3.4	Randomized Controlled Trial; Sample size *n* = 25; Includes sham/BCI control; Evidence strength: high
Ma 2024	EEG	20/20	MI-BCI 40 min + Conventional therapy	Conventional therapy	2 weeks, 10 sessions	FMA-UE	11.2/8.7	Controlled Trial (non-randomized); Sample size *n* = 40; Evidence strength: moderate

AOT, action observational training; ARAT, action research arm test; BCI, brain–computer Interface; BPT, behavioral physical therapy; CG, control group; CON, conventional therapy; CSP, common spatial pattern; EG, experimental group; EES, European stroke scale score; GAS, goal attainment scale; FBCSP, filter bank common spatial pattern; fNIRS, functional near-infrared spectroscopy; FES, functional electrical stimulation; IM, intention of movement; HK, haptic knob; MAL, motor activity long; MAS, modified Ashworth scale; MBI, modified Barthel index; MI, motor imagery; MRC, medical research council; N/A, not available; NDT, neurodevelopmental treatment; SAT, standard activity therapy; SPT, standard physical therapy; TAAM, therapist-assisted arm.

**Table 2:** Study on lower limb functional rehabilitation after stroke based on closed-loop brain-computer interface.

Study	BCI modality	EG/CG	Experimental intervention	Control intervention	Intervention period	Outcome index	Outcome	Quality evaluation
Chung 2015	EEG	5/5	BCI-FES 30 min	FES 30 min	5 day, 5 sessions	TUG BBS Gait functions	BBS: 3.2 ± 2.4/1.6 ± 1.8	Controlled Trial (non-randomized); Sample size *n* = 10; Evidence strength: preliminary
Mrachacz-Kersting 2016	EEG	13/9	CPNS 30–50 times	CPNS-Random 30–50 times	1 week, 3 sessions	FM-LE, 10MWT	FMA: 4.5 ± 2.68/2.57 ± 2.10	Randomized Controlled Trial (RCT); Sample size *n* = 22; Evidence strength: moderate
Sun 2011	EEG	20	Balance training with visual feedback 30 min	NA	4 weeks, 20 sessions	BBS, HWC	FMA: 6–8	Single-group study; Sample size *n* = 20; Evidence strength: preliminary
Sebastián-Romagosa 2023	EEG	22	BCI + MI 60 min	NA	3 months, 25 sessions	FMA-LE, 10MWT, TUG, BBS, FAC	FMA: 4–5	Single-group study; Sample size *n* = 22; Evidence strength: preliminary
Lee 2015	EEG	10/10	Brain wave control stimulation 30 min	Omitting the brain wave control stimulation 30 min	8 weeks, 24 sessions	10MWT	Not numerically reported	Randomized Controlled Trial (RCT); Sample size *n* = 20; Evidence strength: preliminary
McCrimmon 2015	EEG	9	BCI + FES 60 min	NA	4 weeks, 12 sessions	AROM, 6MWD, FM-LM	Not numerically reported	Single-group study; Sample size *n* = 9; Evidence strength: preliminary
Zhao 2022	EEG	14/14	Robot training 30 min, PT training 30 min	EEG recording system not operational, robot training 30 min, PT training 30 min	4 weeks, 24 sessions	FMA-LE, FMA-B, FAC, MBI, BDNF, MEP	FMA: 8–10/4–5	Randomized Controlled Trial (RCT); Sample size *n* = 28; Evidence strength: high
Gao 2022	EEG	5	BCI-MI, visual and motor perception feedback training 15 min	NA	12–14 day, 24–28 sessions	FMA-LE	Not numerically reported	Single-group study; Sample size *n* = 5; Evidence strength: very low
Yuan 2021	EEG	16/14	BCI-PT virtual reality training 1 times	Traditional pedaling	2 weeks, 12 sessions	FMA-LE	Not numerically reported	Randomized Controlled Trial (RCT); Sample size *n* = 30; Evidence strength: high
Colin M. McCrimmon 2014	EEG	3	BCI-FES CPNS 60 min	NA	1 weeks, 3 sessions	AROM	Not numerically reported	Single-group study; Sample size *n* = 3; Evidence strength: very low
Lima 2023	EEG	1	MI-BCI + tDCS 20 min	NA	3 weeks, 15 sessions	FMA-LE Mini BEST	Not numerically reported	Single-group study; Sample size *n* = 1; Evidence strength: very low

FMA-LE, Fugl-Meyer Assessment for Lower Extremity; FMA-B, Fugl-Meyer Assessment for Balance; FAC, Functional Ambulation Category; MBI, Modified Barthel Index; BDNF, brain-derived neurotrophic factor; MEP, motor-evoked potential; AROM, active range of motion; 6MWD, six-minute walk distance; TUG, Timed Up and Go; FES, Functional electrical stimulation; 10MWT, 10 Meter Walking Test; BBS, Berg Balance Scale; CPNS, Common Peroneal Nerve Stimulation; MI, motor imagery; FES, Functional electrical stimulation; HWC, Holden Walking Classification.

**Table 3:** Study on cognitive rehabilitation after stroke based on closed-loop brain-computer interface.

Study	BCI modality	EG/CG	Experimental intervention	Control intervention	Intervention period	Outcome index	Outcome	Quality evaluation
Reichert 2016	EEG	1	SMR training	NA	10 sessions	VLT NVLT	Not numerically reported	Single-group study; Sample size *n* = 1; Evidence strength: preliminary
Kober 2015	EEG	11/6	SMR 12–15 Hz 45 min	NF training 10–12 Hz 45 min	10 sessions	STM LTM WM	Not numerically reported	Controlled trial (non-randomized); Sample size *n* = 17; Evidence strength: preliminary
Tonin 2017	EEG	3	CVSA training	NA	2 weeks, 6 sessions	RT visual paradigm reaction time	Not numerically reported	Single-group Study; Sample size *n* = 3; Evidence strength: very low
Kotov 2019	EEG	22/22	Complex multimodal stimulation	BCI training	NA	Memory Attention Visual constructive skills	Not numerically reported	Controlled Trial (non-randomized); Sample size n = 44; Evidence strength: preliminary
Foong 2020	EEG	11	nBETTER	NA	6 weeks, 18 sessions	Fatigue-monitoring	Not numerically reported	Single-group Study; Sample size *n* = 11; Evidence strength: preliminary
Chung 2020	SMR	13/11	13 BCI-FES 30 min	11FES 30 min	5 weeks, 15 sessions	Executive capacity gait velocity and cadence	Not numerically reported	Randomized Controlled Trial (RCT); Sample size n = 24; Evidence strength: moderate
Sebastián-Romagosa 2020	EEG	51	MI training	NA	13 week, 25 sessions	SCWT MOCA	Not numerically reported	Single-group Study; Sample size n = 51; Evidence strength: preliminary

BCI, brain–computer interface; MI, motor imagery; VLT, verbal learning task; NVLT, non-verbal learning task; STM, short-term memory; LTM, long-term memory; WM, working memory; SWCT, Stroop Color-Word Test; MOCA, Montreal Cognitive Assessment; nBETTER, Neurostyle Brain Exercise Therapy Towards Enhanced; CVSA, Covert visuospatial attention.

## 3 Multi-dimensional classification of brain-computer interfaces

### 3.1 BCI system interaction and feedback control

Based on signal flow, temporal characteristics, and user intention generation, the industry generally categorizes BCIs into three major technical branches: “open-loop/closed-loop,” “synchronous/asynchronous,” and “active/reactive/passive.” (1) Open-loop and closed-loop systems: Open-loop BCIs rely on the brain unidirectionally driving external devices without directly coupled neural sensory feedback; in contrast, closed-loop BCIs drive neural plasticity remodeling at the synaptic level through a “central-peripheral” dual-loop mechanism via real-time sensory feedback (visual, auditory, or proprioceptive). (2) Synchronous and asynchronous systems: Synchronous BCIs (cue-driven) acquire and process brain signals only within specific time windows, whereas asynchronous BCIs (self-paced) allow subjects to generate intentions freely, more closely resembling real-world sensations in natural rehabilitation training. (3) Active, reactive, and passive systems: Active BCIs require the intentional execution of specific cognitive tasks (e.g., motor imagery); reactive BCIs induce specific electrophysiological amplitude changes (e.g., P300 or SSVEP) through external stimuli; passive BCIs can implicitly monitor psychological state changes, such as cognitive workload, fatigue, or emotion, during multitasking (11–13). This study primarily focuses on closed-loop BCIs, with other types serving only as supplementary context.

### 3.2 Closed-loop BCI classification and signal acquisition

Current mainstream approaches are predominantly categorized into invasive, non-invasive, and semi-invasive modalities, with significant differences in detection accuracy and methodology (14). (1) Invasive BCIs typically require implanting microelectrode arrays directly into the cerebral cortex to capture single/multi-neuron action potentials and local field potentials with a high signal-to-noise ratio (SNR) (15–17). In clinical trials involving hemiplegic/tetraplegic patients, these systems have demonstrated advantages in high degrees of freedom and information transfer rates, enabling precise control of external motor prostheses. However, invasive BCIs carry potential risks regarding biocompatibility, infection, and intracranial hemorrhage, which still limit their clinical translation (2, 15, 16, 18). (2) Non-invasive BCIs merely require attachment to the scalp surface, offering high safety as they involve no physiological alterations. Affected by the low-pass filtering properties of the skull and soft tissues, they exhibit limitations in SNR and spatial resolution, often necessitating greater computational costs for background noise reduction and signal acquisition. Benefiting from their superior safety profile, non-invasive BCIs are currently the primary research direction and the preferred choice for implementing stroke rehabilitation (17, 19). (3) Semi-invasive BCIs implant electrode arrays in the subdural or epidural space, significantly reducing the risks of parenchymal damage and infection compared to invasive methods. Simultaneously, they avoid low-pass filtering interference, offering mapping advantages such as high resolution, broad bandwidth (including high-*γ* rhythms), and high spatial overlap. They represent a compromise between signal quality and clinical safety in current BCI technology, suitable for the long-term, stable monitoring of distributed brain networks (20). Furthermore, signal sources can be divided based on biological modalities into electrophysiological and hemodynamic measurements: (1) Electrophysiological signal measurement (EEG/MEG): EEG captures slow cortical potentials and sensorimotor rhythms (SMR, event-related desynchronization [ERD] in the *μ*/*β* bands) from pyramidal neuron synapses. However, due to strong filtering by cranial tissues, its spatial resolution is susceptible to electromyographic (EMG) artifact interference (21). (2) Hemodynamic signal measurement (fMRI/fNIRS): These indirectly measure blood oxygen level-dependent (BOLD) signals reflecting regional brain activity. Functional magnetic resonance imaging (fMRI) provides millimeter-level high spatial resolution across the whole brain but is constrained by bulky equipment and inherent latency, making real-time feedback challenging. Conversely, functional near-infrared spectroscopy (fNIRS) is a portable optical imaging technique that assesses local cortical hemodynamics beneath the skull; however, data utilizing fNIRS in current BCI domains remains sparse (22, 23). Multimodal applications combining fNIRS and EEG are commonly employed to quantitatively evaluate the marginal cortical topological reorganization in the damaged areas before and after closed-loop BCI interventions.

## 4 Applications of closed-loop brain-computer interfaces in stroke rehabilitation

**Figure 2:**
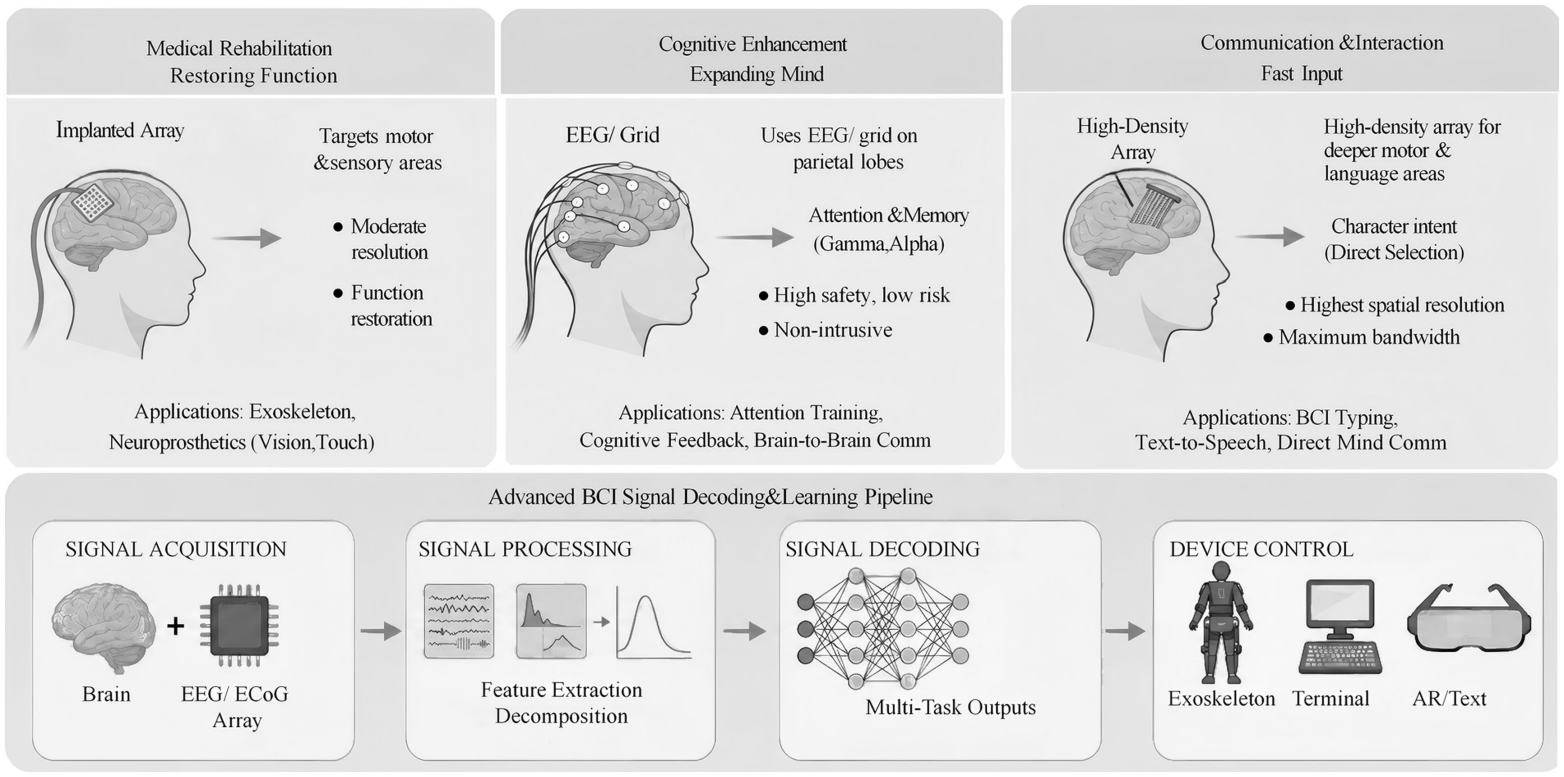
Schematic diagram of closed-loop brain-machine interface. The closed-loop BCI system establishes a bi-directional neural pathway. First, endogenous neural signals (e.g., motor intent) are acquired via non-invasive (EEG) or invasive arrays. Following real-time feature extraction and decoding via machine learning algorithms, the control commands drive external effectors (such as robotic exoskeletons or functional electrical stimulation). Crucially, the subsequent somatosensory and visual feedback is presented back to the patient synchronously with the initial motor intent, thereby promoting Hebbian plasticity and cortical reorganization in the affected hemisphere. EEG, electroencephalography; ECoG, electrocorticography; AR, augmented reality.

The potential therapeutic efficacy of closed-loop BCIs for stroke rehabilitation fundamentally depends on the real-time coupling established between neuronal activity and sensory feedback, involving microscopic changes such as synaptic plasticity and macroscopic neural network reorganization. In exploratory studies involving primate and rodent models, closed-loop BCIs have been shown to induce operant conditioning and Hebbian-like spike-timing-dependent synaptic plasticity (24, 25). This suggests that even in the absence of visual feedback, central neurons can undergo adaptive changes during goal-directed tasks facilitated by the closed-loop system. This provides a crucial translational inspiration for human stroke rehabilitation: by converting EEG signals into interactive commands for external devices, closed-loop BCIs construct a “peripheral-central” closed loop linking endogenous motor intentions with exogenous sensory feedback, utilizing external interventions to drive the functional reorganization of damaged brain regions (26). Although the aforementioned mechanisms predominantly originate from animal models and basic neuroscience research, they furnish an invaluable theoretical foundation for understanding the neuroplastic changes induced by closed-loop BCIs in humans (see Figure 2).

### 4.1 Advances in closed-loop BCIs for upper limb functional rehabilitation after stroke

Addressing the upper limb motor dysfunction that affects approximately 77% of patients in the chronic phase of stroke, closed-loop BCIs offer a passively driven modality for central neural remodeling (27). Based on previous studies, closed-loop interventions for the upper limb can be achieved via two exogenous pathways: first, kinematic traction via devices such as robotic exoskeletons; second, peripheral muscle recruitment based on functional electrical stimulation (FES) (28–35). Although there are differences in models and feedback media between the two (e.g., animal models based on epidural stimulation versus motor imagery training combined with visual/auditory feedback), their underlying logic is consistent—timely coupling of “intention-action”—which may facilitate interhemispheric rebalancing and the reconstruction of the corticospinal tract (36, 37). Synthesizing data from multiple randomized controlled trials utilizing exoskeletons or FES against the MCID threshold criteria, the average improvement in FMA-UE scores for the experimental groups consistently surpassed the 5-point clinical benefit threshold. This comprehensive evidence indicates that closed-loop BCIs yield not only statistical significance in the subacute and chronic phases of stroke but also highlight their potential for yielding clinically meaningful functional improvements (see Table 1).

### 4.2 Advances in closed-loop BCIs for lower limb functional rehabilitation after stroke

In the realm of lower limb motor function reconstruction post-stroke, EEG-based closed-loop BCIs exhibit superior therapeutic efficacy compared to conventional therapies (38). Reviewing the recent development trajectory of closed-loop BCIs, lower limb interventions are evolving from traditional mechanical feedback towards multimodal interventional environments (39, 40). Innovations ranging from EEG-coupled gait training to three-dimensional virtual reality (VR) interactions all point toward the same rehabilitative ideal: high-frequency, precise closed-loop sensory-motor feedback (tactile, visual, and proprioceptive) to systematically stimulate and remodel fundamental neural conduction pathways (41, 42). As posited by McCrimmon et al.’s theoretical framework, multimodal real-time feedback interactions can effectively generalize the ipsilesional sensorimotor cortex and activate motor neurons, which may be associated with the enhanced activation of spinal gait-related neural networks (including Central Pattern Generators, CPGs) (43, 44). In summary, lower limb closed-loop BCIs represent not merely single-device assistance but a systemic multimodal sensory-motor integration therapy. It not only improves walking speed, endurance, and active participation in stroke patients but also provides core clinical evidence for unveiling the complex theoretical mechanisms connecting the central nervous system with peripheral gait networks (see Table 2).

### 4.3 Advances in closed-loop BCIs for cognitive rehabilitation

Cognitive impairment is one of the most common complications following a stroke. Its pathogenic mechanisms involve multiple dimensions, including executive function, memory, and attention, leading to systemic impairments that range from mild cognitive impairment to dementia (45). Multiple memory intervention studies have confirmed that stroke patients undergoing closed-loop BCI neurofeedback training achieve statistically significant improvements in immediate memory, delayed memory, and attentional focus (46–48). This demonstrates that EEG-based neurofeedback training has a positive effect on memory function recovery in stroke patients and helps delay the onset of Alzheimer’s disease and vascular dementia. However, current research evidence remains highly controversial: (1) Insufficient scale sensitivity: In several studies, while closed-loop BCIs showed improvement trends in specific subtests like the SCWT (Stroop Color-Word Test), other conventional scales failed to show statistical significance. The core reason is that existing scales lack the sensitivity to detect fine, higher-order cognitive changes (e.g., executive and attentional networks) mediated by BCIs. (2) Mismatch between closed-loop BCI paradigms and lesion heterogeneity: Mainstream protocols (e.g., MI, P300) predominantly target sensorimotor or visual attention networks, providing limited generalization to other cognitive domains. Furthermore, due to variations in stroke pathology, the same intervention protocol may yield paradoxical results across different individuals (see Table 3) (49). In conclusion, the application of closed-loop BCI technology in post-stroke cognitive rehabilitation is gradually increasing, but broader clinical trials are needed to verify its long-term efficacy and to enable customized adaptations tailored to individual differences, aiming for more robust outcome metrics. Therefore, developing closed-loop BCI training paradigms that target specific cognitive domains remains a crucial direction for future research.

### 4.4 Advances in closed-loop BCIs for speech rehabilitation

Approximately 33.3% of patients present with varying degrees of language impairment post-stroke, including motor aphasia and fluent aphasia (50, 51). Closed-loop BCIs facilitate the restoration of social interaction capabilities for stroke patients by decoding their intentions and interacting with text or speech output devices. A study by Nolfe, G., et al. found a positive correlation between the reduction of P300 amplitude and the severity of aphasia, a discovery that provides an emerging research avenue for BCIs in speech rehabilitation (52). Subsequently, Han, Jin, et al. successfully achieved fluent typing of Chinese characters and English by utilizing a hybrid encoding of P300 and steady-state visual evoked potentials (SSVEP) (53). Furthermore, researchers at Stanford University, building on previous findings, utilized high-performance intracortical BCIs to increase the text output rate by 1.4 to 4.2 times and the information throughput by 2.2 to 4.0 times (54). In addition to enhancing expressive capabilities, BCIs can realize bidirectional communication by decoding, converting, and transmitting EEG signals to another recipient. This implies that even individuals who have completely lost conventional communication skills may eventually achieve information exchange via closed-loop BCI technology (55).

### 4.5 Other application areas

Furthermore, closed-loop BCI technology is currently being applied to the recovery of various post-stroke functional impairments. By monitoring the feedback of swallowing-related brain regions or utilizing neuromodulation techniques, post-stroke dysphagia has been substantially alleviated, significantly improving the precision of swallowing in affected patients (56). For patients with impaired visual systems following a stroke, visual devices (e.g., navigation systems or visual aids) are used to translate external light signals into electrical signals transmitted to brain regions, thereby expanding the patients’ range of activities and enhancing their activities of daily living (ADL) (57). Additionally, closed-loop BCI technology is exploring various cerebral and psychological therapies, having already demonstrated preliminary success (58). Consequently, the referenceable treatment protocols brought forth by closed-loop BCIs offer diverse and personalized options for stroke patients.

## 5 Neural remodeling mechanisms of closed-loop BCIs in stroke rehabilitation

By integrating real-time neural signal decoding with precise exogenous feedback, closed-loop BCIs can trigger neuroplastic remodeling across both the microscopic synaptic and macroscopic network dimensions (59). In neuroprosthetic control models, the brain’s control reconfiguration is not restricted to the primary motor cortex (M1); it also involves higher-order motor areas and association cortices (24). Concurrently, neuronal activity in cortical regions such as the striatum and cerebellum plays a critical endogenous gating role in BCI skill acquisition, consolidation, and behavioral fine-tuning. This distributed brain network synergy establishes the anatomical foundation for central lesion functional compensation and network reconstruction (60). Under this global network architecture, closed-loop BCIs microscopically intercept endogenous “motor intentions” and exogenous “sensory feedback,” achieving millisecond-level synchronous coupling. Closed-loop BCI interventions based on motor imagery target and activate the damaged hemispheric motor areas, inducing synaptic changes at the micro-level. This promotes the formation of new synaptic connections and enhances the recruitment of local neurons in the perilesional zones (61). Meanwhile, retrograde sensory afferents generated by external robots or functional electrical stimulation (FES) augment the metabolic activity of targeted brain regions, expanding the structural and functional reorganization space of the local cortex through “central-peripheral” retrograde feedback loops (62).

Beyond promoting focal cortical remodeling, closed-loop BCIs also participate in the systemic regulation of interhemispheric reciprocal inhibition balance. Due to unilateral focal cortical lesions, the pre-existing interhemispheric inhibitory equilibrium is disrupted, characterized by excessive inhibition from the affected hemisphere and abnormal excitability of the unaffected hemisphere. This pathological asymmetric electrophysiological characteristic is a major mechanism hindering the restoration of voluntary movement in the paretic limb (63). Conversely, closed-loop BCIs capture specific targeted EEG rhythms (e.g., ERD in the *μ*/*β* bands) from the affected hemisphere in real-time, instantly converting them into positive exogenous feedback. This selectively drives the upregulation of excitability in the affected cortex, attenuates the abnormal transcallosal inhibition from the intact hemisphere, and gradually restores the electrophysiological symmetry between the bilateral cortices (64, 65). Neuroimaging and electrophysiological assessments indicate that BCI-robot interventions enhance the feedforward and feedback functional connectivity between the prefrontal and primary motor cortices, with the increase in connection density linearly correlating with limb motor scores. Reports on descending motor-evoked potentials (MEPs) show that following closed-loop BCI rehabilitation training, the conduction amplitude of the corticospinal tract in the affected hemisphere is progressively upregulated (66). This indirectly corroborates the positive involvement of closed-loop BCIs in enhancing corticospinal pathway conduction, accomplishing the precise translation of neural motor intentions into physical limb movements (67).

## 6 Adverse reactions and safety evaluation

The safety evaluation of closed-loop BCIs based on different acquisition modalities has gradually accumulated, generally demonstrating an absence of severe adverse reactions overall (68). Invasive BCIs: Long-term follow-ups of human cohorts (e.g., the BrainGate system with over 12,000 device-days) show that the incidence of device-related adverse events is approximately 5.6 per 1,000 device-days, with serious adverse events around 0.49 per 1,000 device-days. The most common events are localized skin reactions around the electrodes; no related intracranial infections, permanent disabilities, or deaths have been observed, and there is no evidence attributing epileptic activity to the devices (69). Semi-invasive BCIs (subdural/epidural): Their risk profile falls between invasive and non-invasive methods. The infection risk for subdural electrodes is approximately 1–3%, and the risk of symptomatic hemorrhage is 0–4% (influenced by the surgical center’s experience and monitoring duration), with permanent neurological deficits occurring very rarely (<1%) (70). Non-invasive BCIs (EEG/fNIRS): These present no significant adverse reactions, mostly manifesting as mild headaches, visual or cognitive fatigue, and occasionally localized skin erythema when combined with surface FES, all of which typically resolve spontaneously after 15–20 min of rest (see Table 4) (71). Currently, there is a lack of externally validated clinical prediction models for individualized BCI surgical/device complications; thus, it is imperative to leverage frameworks like CONSORT-AI to build multimodal risk early-warning systems.

**Table 4:** Summary of device-related adverse events in closed-loop BCI trials.

BCI modality	Mild adverse events (incidence)	Serious adverse events (incidence)
Invasive BCI	Skin irritation (~5.6/1,000 device-days)	None reported for infection or death (overall ~0.49/1,000 device-days)
Semi-invasive BCI	Infection (1–3%), symptomatic hemorrhage (0–4%)	Permanent neurological deficits (<1%)
Non-invasive BCI	Headache, fatigue, occasional skin erythema	None reported

Brain-computer interface. Non-invasive BCI adverse events are typically intensity-dependent and resolve rapidly with rest optimization.

### 6.1 Limitations

Despite the optimistic prospects of closed-loop BCIs in stroke rehabilitation, several methodological and technological challenges remain. Firstly, approximately 15 to 30% of subjects are unable to produce stably decodable EEG rhythms (e.g., the ERD/ERS phenomena). This is particularly pronounced in stroke patients with damaged brain networks, which severely restricts its universal applicability (72). Secondly, current studies are characterized by small sample sizes, and subjects often perceive a mismatch between the feedback signals and their endogenous intentions, making it impossible to completely rule out potential placebo effects (73, 74). Finally, although closed-loop BCIs combined with machine learning perform exceptionally well in laboratory settings, their robustness and comfort in complex real-world environments remain inadequate. Future endeavors must address the challenge of individual model generalization while simultaneously improving systemic stability (75). We have yet to fully comprehend the intricate neural mechanisms of the brain, making it difficult to leverage these mechanisms to refine closed-loop BCI technology. Although human-machine interaction has made significant strides, technological upgrades and ethical frameworks regarding information transfer rates and privacy protection require further fortification (76).

## 7 Conclusion and perspectives

**Figure 3:**
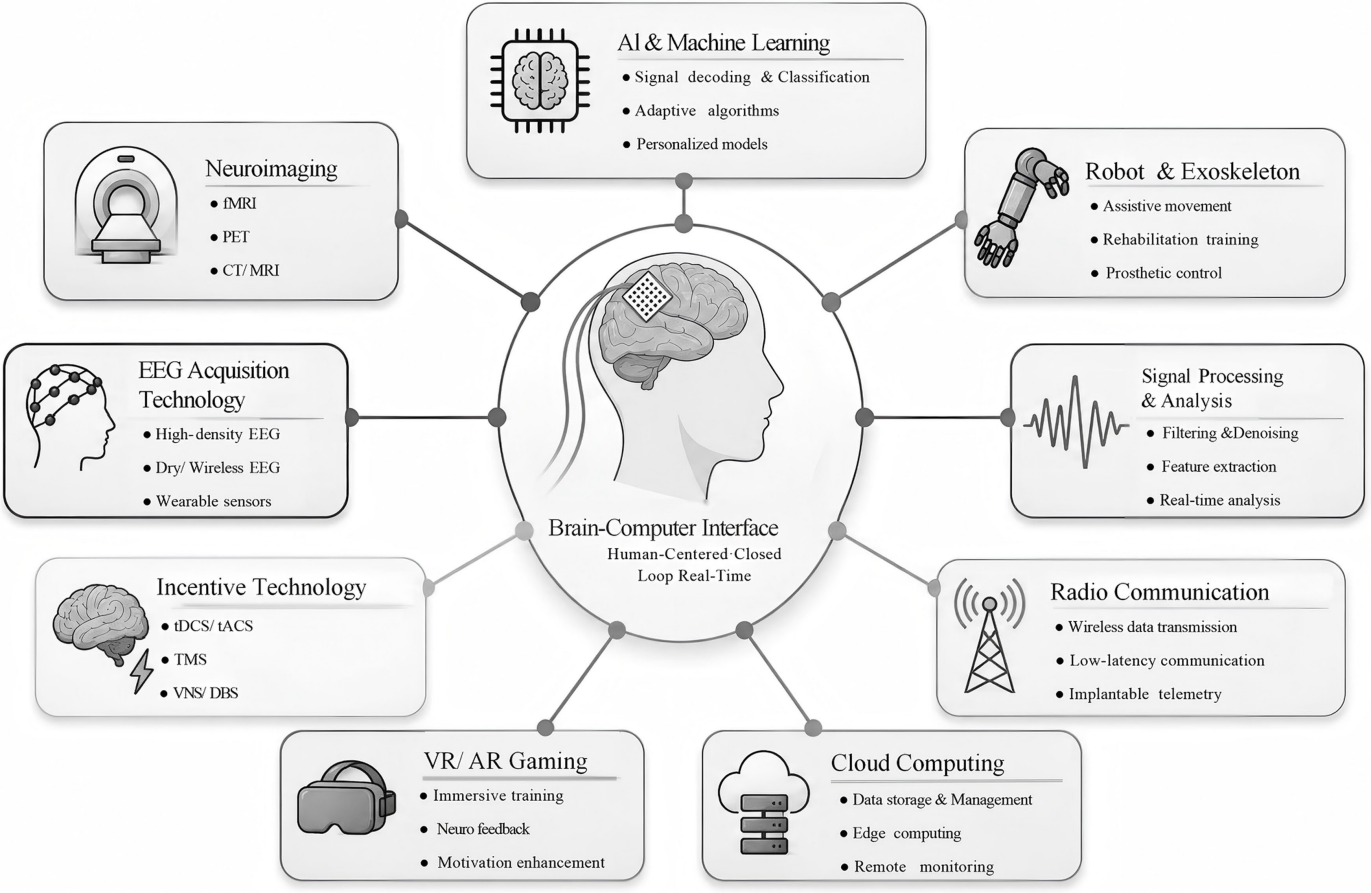
Classification, applications, development, conclusions and perspectives of closed-loop BCIs. The next generation of BCI-based stroke rehabilitation relies on a human-centered, multi-modal ecosystem. This includes the integration of neuroimaging (e.g., fMRI) for precise anatomical targeting, advanced AI and machine learning for adaptive decoding and personalized modeling, and cloud computing for multi-center federated learning. Furthermore, combining incentive technologies (such as tDCS and TMS) with VR/AR environments can synergistically enhance patient motivation and cortical excitability. fMRI, functional magnetic resonance imaging; tDCS, transcranial direct current stimulation; TMS, transcranial magnetic stimulation.

BCIs trigger neuroplastic remodeling at both microscopic synaptic and macroscopic network levels by integrating real-time neural signal decoding with exogenous feedback. In fact, closed-loop BCIs rely not only on the complex motor coordination functions of the brain network to activate the damaged primary motor cortex and higher-order association areas but also extensively recruit crucial nuclei in the striatum and cerebellum to exert endogenous gating. Under this global network synergy, endogenous motor intentions and sensory feedback form a millisecond-window coupling, which induces synaptic reconstruction and metabolic upregulation in the damaged cortex, rectifying the pathological electrophysiological abnormalities of the bilateral hemispheres. Furthermore, imaging and electrophysiological evidence demonstrates that the feedforward and feedback motor potentials of the sensorimotor cortex are upregulated post-intervention, enhancing corticospinal tract conduction. However, closed-loop BCIs still face challenges regarding detection accuracy, user comfort, and decoding computational power, necessitating a shift toward standardization and intelligence. In terms of clinical trial design, large-sample, multicenter randomized controlled trials stratified by lesion location and clinical stage are urgently needed to establish a core outcome measure set that includes functional (e.g., FMA-UE, 10MWT), cognitive-linguistic (e.g., MoCA), neurophysiological (e.g., ERD, TMS), and standardized safety assessments (77–79). Concurrently, closed-loop BCIs hold promise for deep integration with artificial intelligence: leveraging autonomous learning to strengthen decoding capabilities and optimizing individualized stimulation parameters, thereby offering more diverse and personalized customized options for stroke patient rehabilitation (see Figure 3) (80).
