# Supplementary figures and images for: Advancing stroke rehabilitation: the potential and challenges of closed-loop brain-computer interface technology
Source: Front Neurol. 2026 Jun 24;17:1861673. doi: 10.3389/fneur.2026.1861673 (PMC13341518; doi:10.3389/fneur.2026.1861673)

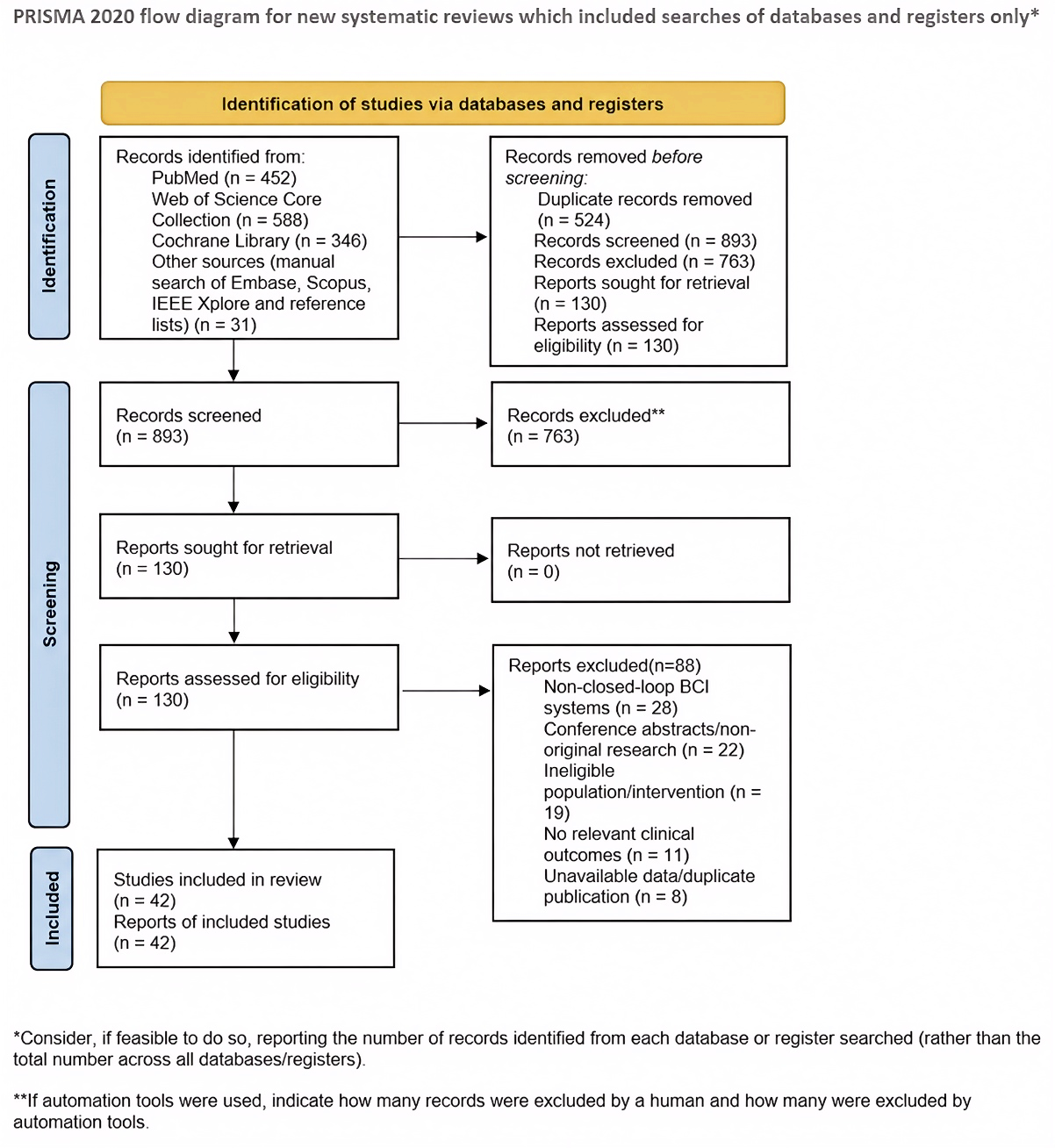

Supplement: Supplementary file 1 [file Image_1.PNG]
